# A proteogenomic analysis of the adiposity colorectal cancer relationship identifies GREM1 as a probable mediator

**DOI:** 10.1093/ije/dyae175

**Published:** 2025-01-22

**Authors:** Matthew A Lee, Charlie A Hatcher, Emma Hazelwood, Lucy J Goudswaard, Konstantinos K Tsilidis, Emma E Vincent, Richard M Martin, Karl Smith-Byrne, Hermann Brenner, Iona Cheng, Sun-Seog Kweon, Loic Le Marchand, Polly A Newcomb, Robert E Schoen, Ulrike Peters, Marc J Gunter, Bethany Van Guelpen, Neil Murphy

**Affiliations:** International Agency for Research on Cancer (IARC/WHO), Nutrition and Metabolism Branch, Lyon, France; Population Health Sciences, Bristol Medical School, University of Bristol, Bristol, United Kingdom; Population Health Sciences, Bristol Medical School, University of Bristol, Bristol, United Kingdom; Medical Research Council Integrative Epidemiology Unit, University of Bristol, Bristol, United Kingdom; Population Health Sciences, Bristol Medical School, University of Bristol, Bristol, United Kingdom; Medical Research Council Integrative Epidemiology Unit, University of Bristol, Bristol, United Kingdom; Population Health Sciences, Bristol Medical School, University of Bristol, Bristol, United Kingdom; Medical Research Council Integrative Epidemiology Unit, University of Bristol, Bristol, United Kingdom; Department of Epidemiology and Biostatistics, School of Public Health, Imperial College London, London, United Kingdom; Department of Hygiene and Epidemiology, University of Ioannina School of Medicine, Ioannina, Greece; Population Health Sciences, Bristol Medical School, University of Bristol, Bristol, United Kingdom; Medical Research Council Integrative Epidemiology Unit, University of Bristol, Bristol, United Kingdom; School of Translational Health Sciences, University of Bristol, Bristol, United Kingdom; Population Health Sciences, Bristol Medical School, University of Bristol, Bristol, United Kingdom; Medical Research Council Integrative Epidemiology Unit, University of Bristol, Bristol, United Kingdom; National Institute for Health Research (NIHR) Bristol Biomedical Research Centre, University Hospitals Bristol and Weston NHS Foundation Trust and the University of Bristol, Bristol, United Kingdom; Cancer Epidemiology Unit, University of Oxford, Oxford, United Kingdom; Division of Clinical Epidemiology and Aging Research, German Cancer Research Center (DKFZ), Heidelberg, Germany; Division of Preventive Oncology, German Cancer Research Center (DKFZ) and National Center for Tumor Diseases (NCT), Heidelberg, Germany; German Cancer Consortium (DKTK), German Cancer Research Center (DKFZ), Heidelberg, Germany; Department of Epidemiology and Biostatistics, University of California-San Francisco, San Francisco, CA, United States; Department of Preventive Medicine, Chonnam National University Medical School, Gwangju, Korea; Jeonnam Regional Cancer Center, Chonnam National University Hwasun Hospital, Hwasun, Korea; University of Hawaii Cancer Center, Honolulu, HI, United States; Public Health Sciences Division, Fred Hutchinson Cancer Center, Seattle, WA, United States; Department of Epidemiology, University of Washington, Seattle, WA, United States; Department of Medicine and Epidemiology, University of Pittsburgh Medical Center, Pittsburgh, PA, United States; Public Health Sciences Division, Fred Hutchinson Cancer Center, Seattle, WA, United States; Department of Epidemiology, University of Washington, Seattle, WA, United States; International Agency for Research on Cancer (IARC/WHO), Nutrition and Metabolism Branch, Lyon, France; Department of Epidemiology and Biostatistics, School of Public Health, Imperial College London, London, United Kingdom; Department of Diagnostics and Intervention, Oncology, Umeå University, Umeå, Sweden; Wallenberg Centre for Molecular Medicine, Umeå University, Umeå, Sweden; International Agency for Research on Cancer (IARC/WHO), Nutrition and Metabolism Branch, Lyon, France

**Keywords:** adiposity, proteome, colorectal cancer, Mendelian randomization, colocalization

## Abstract

**Background:**

Adiposity is an established risk factor for colorectal cancer (CRC). The pathways underlying this relationship, and specifically the role of circulating proteins, are unclear.

**Methods:**

Utilizing two-sample univariable Mendelian randomization (UVMR), multivariable Mendelian randomization (MVMR), and colocalization, based on summary data from large sex-combined and sex-specific genetic studies, we estimated the univariable associations between: (i) body mass index (BMI) and waist–hip ratio (WHR) and overall and site-specific (colon, proximal colon, distal colon, and rectal) CRC risk, (ii) BMI and WHR and circulating proteins, and (iii) adiposity-associated circulating proteins and CRC risk. We used MVMR to investigate the potential mediating role of adiposity- and CRC-related circulating proteins in the adiposity–CRC association.

**Results:**

BMI and WHR were positively associated with CRC risk, with similar associations by anatomical tumor site. In total, 6591 adiposity–protein (2628 unique circulating proteins) and 33 protein–CRC (7 unique circulating proteins) associations were identified using UVMR and colocalization. One circulating protein, GREM1, was associated with BMI (only) and CRC outcomes in a manner that was consistent with a potential mediating role in sex-combined and female-specific analyses. In MVMR, adjusting the BMI–CRC association for GREM1, effect estimates were attenuated—suggestive of a potential mediating role—most strongly for the BMI–overall CRC association in women.

**Conclusion:**

Results highlight the impact of adiposity on the plasma proteome and of adiposity-associated circulating proteins on the risk of CRC. Supported by evidence from UVMR and colocalization analyses using *cis*-single-nucleotide polymorphisms, GREM1 was identified as a potential mediator of the BMI–CRC association, particularly in women.

Key Messages

- We investigated whether circulating proteins are mediators of the association between body mass index (BMI) and waist–hip ratio with sex- and site-specific colorectal cancer (CRC).
- Using univariable and multivariable Mendelian randomization in combination with colocalization, we identified a single protein (GREM1; out of 4907) that showed evidence of mediating the effect of BMI in the association with CRC in women.
- GREM1 is an adipokine with multiple studies linking its expression to CRC including evidence that a locus interacts with BMI in the positive association with CRC.

## Introduction

Adiposity is an established causal risk factor for the development of colorectal cancer (CRC).^1–3^ However, the underlying biological pathways are incompletely understood. Identifying potentially modifiable mediators of this relationship could uncover targets for pharmacological and/or lifestyle intervention. Evidence from molecular epidemiological and genetic studies has linked adiposity with broad changes in the human circulating proteome, including via effects on metabolism and inflammatory and immune markers.^4–7^ Whether adiposity-associated changes to the proteome influence the association between adiposity and CRC risk is unclear.

Mendelian randomization (MR) uses genetic variants as instrumental variables that, under specific assumptions, can be used to investigate causal relationships. Given the random allocation of alleles during gametogenesis, across a large enough population, findings of MR analyses are more robust to the effects of confounding and reverse causation than those of traditional observational studies. Two-step/network MR^8^^,^^9^ and multivariable MR (MVMR)^10^ analyses can be used to investigate traits as intermediates.^11^ To date, MVMR analyses examining the role of the proteome as an intermediate in the relationship between adiposity and CRC have not been undertaken.

We investigated whether circulating proteins act as intermediates between measures of overall adiposity (body mass index; BMI) and abdominal/subcutaneous adiposity (waist–hip ratio; WHR) and CRC risk. We conducted colocalization and MVMR analyses using summary statistics from genome-wide association studies (GWAS) of adiposity traits, circulating proteins, and CRC risk, and examined whether mediating proteins were expressed in adipose and CRC tissue.

## Methods

All analyses were performed using R version 4.1.2 and the following packages: TwoSampleMR^12^ (version 0.4.22), MVMR^13^ (version 0.3), and coloc^14^ (version 5.2.0). Forest plots were created using ggforestplot (version 0.1.0).

### Study design

**Figure 1.**
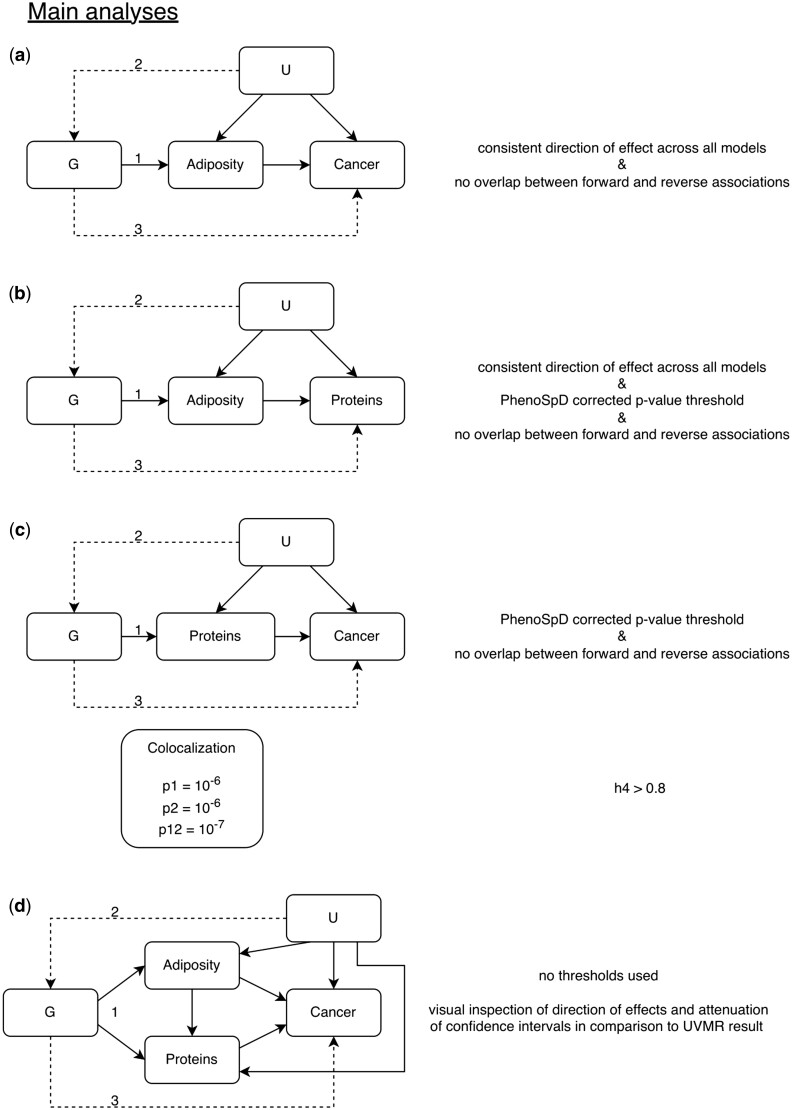
Analysis overview. Directed acyclic graph overview of main analyses. i–iii: univariable Mendelian randomization (MR) analyses, iv: multivariable MR analysis. Text to the right of each analysis gives the requirements for an association. MR assumptions: (1) the instrument is associated with the exposure, (2) there are no confounders of the association between the instrument and the outcome, (3) the instrument is not related to the outcome except via its effect on the exposure; G: genetic variant(s); U: unmeasured confounders; p*: prior probability of a random single nucleotide polymorphism in the region (1) being (causally associated with Trait 1 and not Trait 2, (2) Trait 2 and not Trait 1, or (12) both traits; h4: probability that there is an association with both traits in the region (shared causal variant)

Four main analyses were performed (Fig. 1) to estimate the causal relationship between: (i) adiposity measures (BMI, WHR^8^) and CRC risk, (ii) adiposity measures and circulating proteins, (iii) circulating proteins and CRC risk, and (iv) the mediating effects of adiposity-associated circulating proteins in the adiposity–CRC association. We performed forward and reverse univariable MR (UVMR) for Steps i–iii and used MVMR for Step iv. For Step iii, we performed *cis*-single-nucleotide polymorphism (*cis*-SNP) UVMR and colocalization. For all steps, sex-combined and sex-specific analyses were performed.

### Data sources and study populations

Details of the data sets, study populations, and thresholds that were used in these analyses are available in Supplementary Tables S1 and S2. (See online supplementary material for color versions of these tables.) Data for adiposity measures (European ancestries; sex-combined and sex-specific) were obtained from Pulit *et al.* (2019);^15^ BMI and WHR were derived from self-reported and clinical measures of height and weight and waist and hip circumference, and calculated as $\mathrm{BMI}=\frac{\mathrm{weight}\;\left(kg\right)}{{\left(\mathrm{height}\;\left(m\right)\right)}^{2}}$ and $\mathrm{WHR}=\frac{\mathrm{waist}\;\mathrm{circumference}\;\left(cm\right)}{\mathrm{hip}\;\mathrm{circumference}\;\left(cm\right)}$. Data for CRC (European and East Asian ancestries; sex-combined and sex-specific) were obtained from Huyghe *et al.* (2019);^16^ these data have been reported previously^2^^,^^17^ as physician-diagnosed and we make the assumption from the supplementary data of Huyghe *et al.* that these are predominantly incident cases of unknown stage; DNA samples and data were provided prior to or shortly after diagnosis. Data for ≤4907 aptamers from Ferkingstad *et al.* (2021)^18^ (Icelandic population; sex-combined) were measured in ethylenediaminetetraacetic acid plasma samples using SomaScan^®^ (SomaLogic, v4). Separate SOMAmers can bind to isoforms of the same circulating protein and at different sites (which can be impacted by post-translational modifications or protein complexes formed with other circulating proteins) enabling a larger number of circulating proteins and circulating protein complexes to be quantified.^19^

For all MR analyses, a minimum genome-wide significance threshold of *P* = 5 × 10^−8^ was used for all data (more stringent thresholds were used for adiposity measures and circulating proteins given wider genotyping coverage^20^) and a linkage disequilibrium (LD) independence threshold of 0.001 was used where applicable (Supplementary Table S1). (See online supplementary material for a color version of this table.) For all exposures, F-statistics were calculated for each SNP and a mean was calculated for each instrument, with an F-statistic of >10 indicating a strong instrument.^21^ Circulating proteins were included in two UVMR analyses: *cis*- and *trans*-SNPs were used for reverse MR analyses of the association between adiposity measures and circulating proteins to examine reverse causation; *cis*-SNPs were used in the forward MR analyses of the association between circulating proteins and CRC.

*cis*-SNPs were obtained directly from Ferkingstad *et al.* and were defined as SNPs reaching the genome-wide significance threshold (*P* < 1.8 × 10^−9^), which were ≤1 mega base (Mb) from the transcription start site of the protein-coding gene. Clumping, to remove SNPs highly correlated with the lead SNP through LD, was performed around the SNP with the lowest *P*-value within the 1-Mb region until no overlapping regions remained. In total, 1490 of 4907 aptamers had *cis*-SNPs.

Data for adiposity and circulating proteins were inverse rank normally transformed prior to genome-wide analysis. Assuming that the distribution of each trait was normal prior to transformation and genome-wide analysis, we interpret these units to be approximately equivalent to a normalized standard deviation (SD) of the respective trait. Estimates and odds ratios (ORs) are interpreted as the change in outcome per normalized SD unit change in the exposure.

### Statistical analysis

MR relies upon three core assumptions: (i) the instrument is associated with the exposure, (ii) there are no confounders of the association between the instrument and the outcome, and (iii) the instrument is not related to the outcome except via its effect on the exposure. The same assumptions are extended to include the intermediate in MVMR: (i) the instrument is associated with the exposure given the presence of the mediator, (ii) the instrument is independent of the outcome given both exposure and mediator, and (iii) the instrument is not related to the outcome except via its effect on the exposure given the presence of the mediator. Assumption (i) may be satisfied by using a standard genome-wide significance threshold of 5 × 10^−8^ and instruments with an F-statistic, or conditional F-statistic for MVMR,^22^ of >10. Assumptions (ii) and (iii) are unverifiable but were tested by using models sensitive to the effects of pleiotropy and with colocalization. Colocalization attempts to differentiate between distinct causal variants (which likely result from LD) and a single shared signal^20^ and can be used to assess the validity of MR assumptions and strengthen evidence for a causal effect.^23^

#### Identification of associations

An adiposity–CRC association (Step i) was identified if there was a consistent direction of effect across all models and there was no consistent direction of effect in the reverse MR analyses. The same requirement plus a PhenoSpD^24–26^ (pheno spectral decomposition) corrected *P*-value threshold (we used the more conservative of the two approaches applied by PhenoSpD) was used to identify adiposity–protein associations (Step ii). A protein–CRC association (Step iii) was identified if the PhenoSpD corrected *P*-value threshold was met, there was no consistent direction of effect across all MR models in the reverse MR, and evidence of colocalization (h4 ≥ 0.8) was observed. We interpreted a circulating protein as having a potential mediating role in the adiposity and CRC relationship if the MVMR result adjusting for that circulating protein attenuated towards the null when compared with the UVMR result (Step iv). We performed PhenoSpD on all 4907 proteins and identified a total of 1293 independent variables (*P* = 3.97 × 10^−5^).

#### Univariable Mendelian randomization

Where more than one SNP was available for an exposure, an inverse-variance weighted (IVW), multiplicative random-effects (IVW-MRE) model was used. The model assumes that the strength of the association of the genetic instruments with the exposure is not correlated with the magnitude of the pleiotropic effects and that the pleiotropic effects have an average value of zero.^27^ Where only one SNP was present, the Wald ratio was used. Where genetic variants were not available in the outcome GWAS, proxy SNPs were included if LD was ≥0.8.

##### Sensitivity analysis

The assumptions of no pleiotropy among genetic instruments and outcomes were explored using MR–Egger-,^28^ weighted median-,^29^ and weighted mode^30^-based estimators where at least three SNPs were available. MR–Egger provides an estimate of unbalanced or directional horizontal pleiotropy via the intercept of a linear regression of the SNP–exposure and SNP–outcome association.^28^ The weighted median provides consistent estimates when ≥50% of included instruments are invalid.^29^ The weighted mode assumes that the true causal effect is the most common effect and it is robust when most effect estimates are derived from valid instruments.^30^ In addition, sensitivity analyses using single-SNP (Wald ratio) and ‘leave-one-out’ MR analyses assessed the influence of individual variants on the observed associations. We performed Steiger directionality tests^27^ to assess whether variants used in the analysis of adiposity measures and CRC might be biased by reverse causation; CRC prevalence information for the UK and Europe (excluding the UK) were obtained from the Global Cancer Observatory (https://gco.iarc.who.int; accessed 2 July 2024).

#### Colocalization

For each circulating protein, the *cis*-SNP was extracted along with a 1-Mb window. This region was then extracted from each CRC GWAS and colocalization was implemented using the single causal variant approach.^31^ The LD matrix was generated using the 1000 genomes reference panel (Phase 3) and priors were set at p^1^ = 10^−6^, p^2^ = 10^−6^, and p^12^ = 10^−7^ based on a window of 5000 SNPs (https://chr1swallace.shinyapps.io/coloc-priors/, accessed 15 May 2023). Sensitivity analyses were performed using windows of 250 kb, 500 kb, and 2 Mb, and the following sets of priors: (i) p^1^ = 10^−5^, p^2^ = 10^−5^, p^12^ = 10^−6^; (ii) p^1^ = 10^−6^, p^2^ = 10^−6^, p^12^ = 10^−7^; (iii) p^1^ = 10^−6^, p^2^ = 10^−6^, p^12^ = 10^−7^. We considered evidence of colocalization to be strongest when observed across multiple windows and sets of priors.

#### Multivariable Mendelian randomization

SNPs (*cis*- and *trans*-SNPs) associated with adiposity measures and proposed intermediate circulating proteins were extracted and combined. These SNPs were extracted from the adiposity GWAS and clumped to remove duplicate SNPs and SNPs in LD with one another using the same thresholds as with the UVMR analysis. An IVW model was used to obtain the direct causal effect of each adiposity measure adjusted for each circulating protein on CRC risk. Instrument strength was estimated using a generalized version of Cochran’s Q^10^ assuming a pairwise covariance of zero.^13^

#### Protein expression analyses

To investigate whether circulating proteins included in the MVMR analyses were expressed in adipose and CRC tissue, we used GTEx^32^ (v8) to compare protein-coding gene expression in tissue relative to whole blood using the Wilcoxon rank sum test and visualized using violin plots.

## Results

Supplementary data are available from Zenodo at https://zenodo.org/record/7780822#.ZCQ3U-xBz0o, including a STROBE-MR checklist.^33^^,^^34^

### Association between adiposity and colorectal cancer

**Figure 2.**
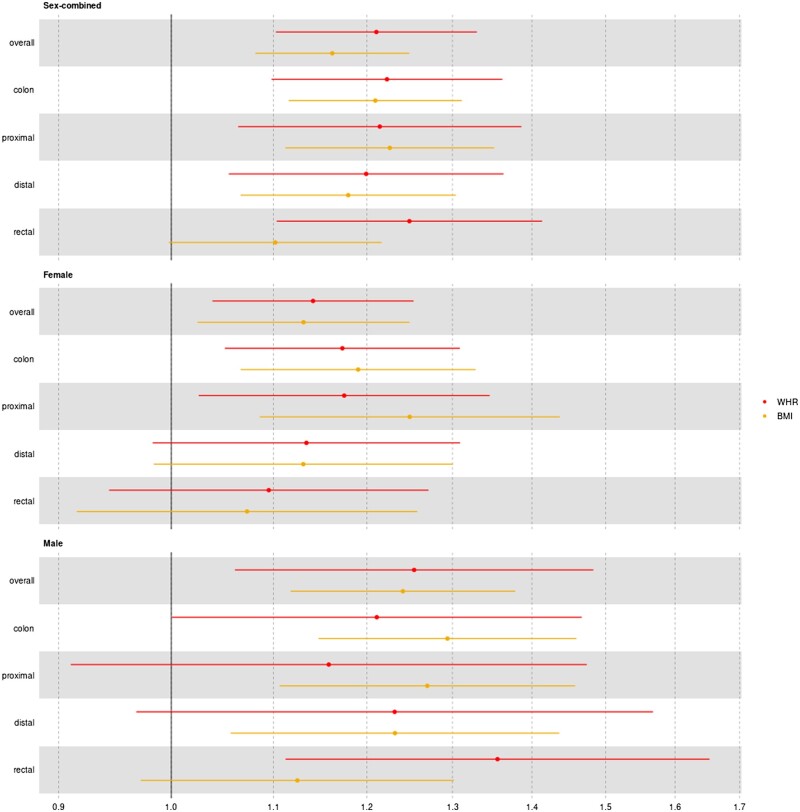
Association between adiposity measures and colorectal cancer outcomes. Odds ratios and 95% confidence intervals shown for the main analysis using the inverse-variance weighted multiplicative random-effects (IVW-MRE) model. BMI, body mass index; WHR, waist–hip ratio

In Step i, BMI and WHR were positively associated with overall and site-specific CRC risk in men and women (Fig. 2); sensitivity models were broadly consistent except for associations between WHR and distal colon cancer and overall CRC in men and rectal cancer in women (Supplementary Fig. S1). (See online supplementary material for a color version of this figure.) The reverse UVMR analyses (Supplementary Fig. S2) showed an increasing effect of proximal and distal colon cancer on WHR that was consistent in sensitivity analyses. (See online supplementary material for a color version of this figure.) Steiger directionality tests indicated the true causal direction was being tested for all adiposity–CRC and CRC–adiposity analyses (Supplementary Table S9). (See online supplementary material for a color version of this table.) The pairings of WHR and proximal and distal colon cancer were excluded from further analyses.

### Association between adiposity and proteins

**Figure 3.**
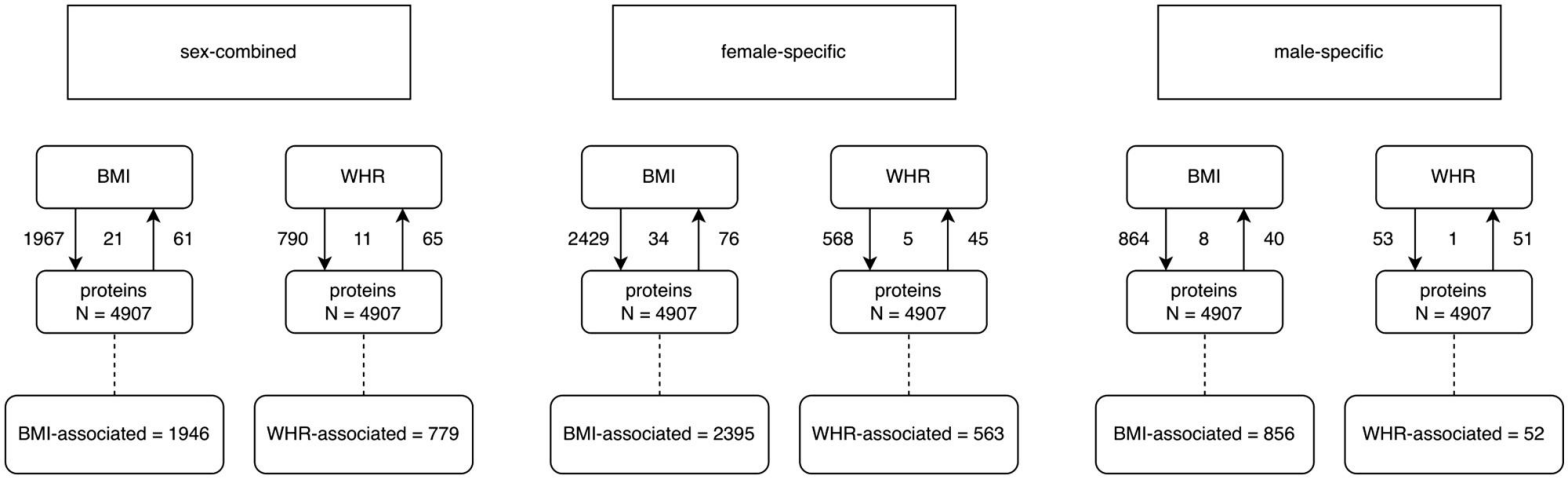
Overview of associations between adiposity measures and circulating proteins (Step ii in the main analysis plan). Arrows show the direction of the univariable (UV) Mendelian randomization (MR) (UVMR) analysis. Values on the outside of the lines indicate the number of associations identified in that direction; values between the lines indicate the number of associations identified in both directions and for which there is, therefore, conflicting evidence of association. Associations were identified if all MR models had consistent directions of effect and a PhenoSpD (pheno spectral decomposition) corrected *P*-value (3.97 × 10^−5^) was met for the main MR model. *N* gives the number of circulating proteins available for analysis. BMI-WHR-associated gives the number of circulating proteins with unconflicted evidence of association with the adiposity measure. BMI, body mass index; WHR, waist–hip ratio

**Figure 4.**
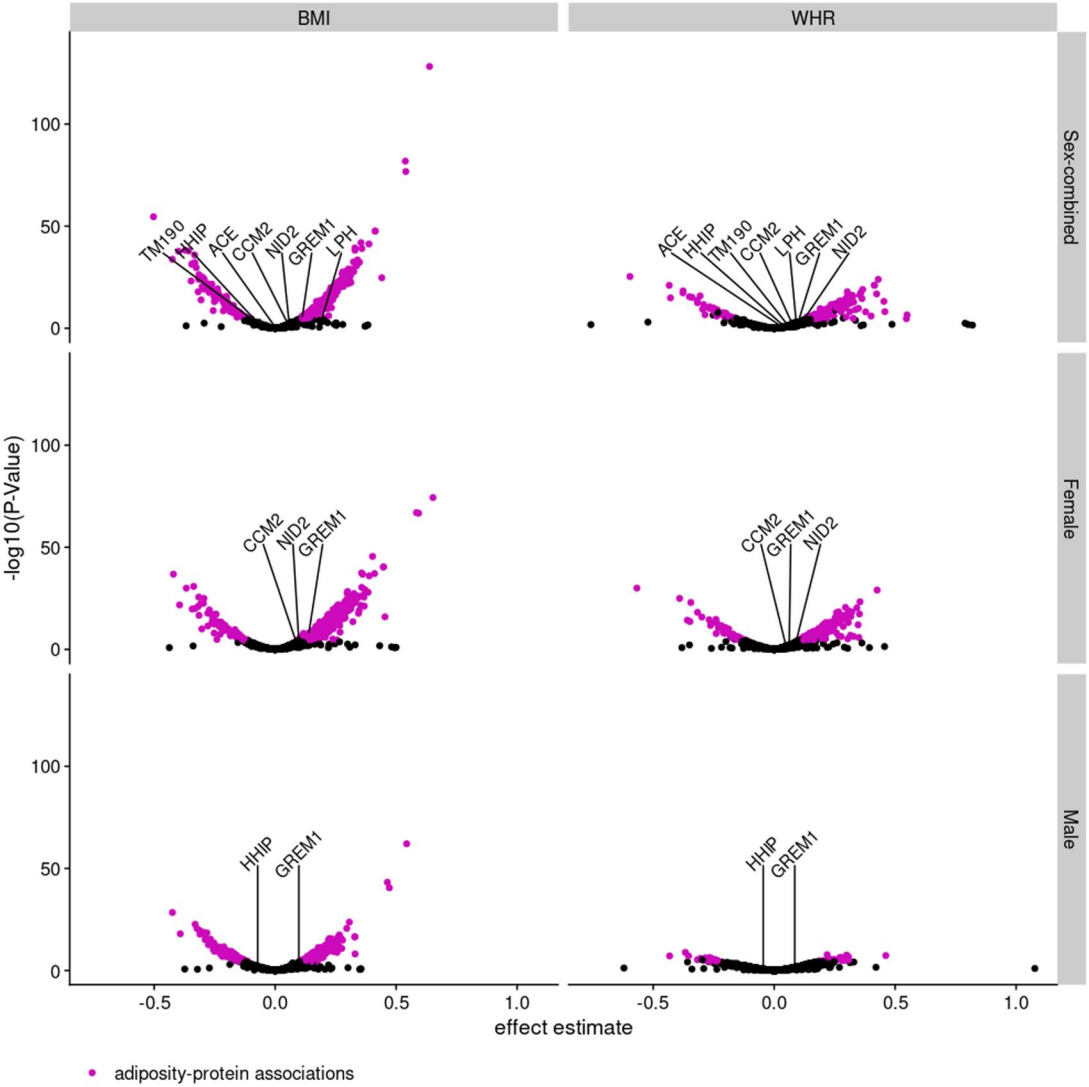
Association between adiposity measures and circulating proteins in Step ii of the main analysis plan. The volcano plot shows effect estimates and -log10(P-val). Adiposity–protein associations are highlighted [analyses reaching the PhenoSpD (pheno spectral decomposition) corrected *P*-value (0.05/1293)], consistent directions of effect across Mendelian randomization (MR) models, and no conflicting association identified in the reverse univariable (UV) MR. Proteins labeled by name were associated with both adiposity and colorectal cancer outcomes, the latter as determined in the UVMR analysis in Step iii of the main analysis plan. BMI, body mass index; WHR, waist–hip ratio

In Step ii, we investigated the association between BMI and WHR with 4907 circulating proteins. Across all analyses, 6591 adiposity–protein associations were identified (Figs 3 and 4, and Supplementary Table S5). (See online supplementary material for a color version of this table.) Of these 6591 adiposity–protein associations, 2628 unique circulating proteins were associated with at least one sex-specific adiposity measure. For example, in sex-combined analysis of BMI, 1967 BMI–protein associations and 61 protein–BMI associations were identified, 21 of these associations overlapped leaving 1946 proteins with unconflicted associations (Fig. 3), and these BMI–protein pairs were taken forward. Figure 4 presents the effect estimates and *P*-values for the forward (adiposity–protein) MR results. Adiposity-associated circulating proteins indicated in the boxes at the bottom of Fig. 3 are highlighted in Fig. 4. Among these, circulating proteins also associated with CRC risk (step iii) are labeled by name (based on results in Fig. 6).

### Association between proteins and colorectal cancer

**Figure 5.**
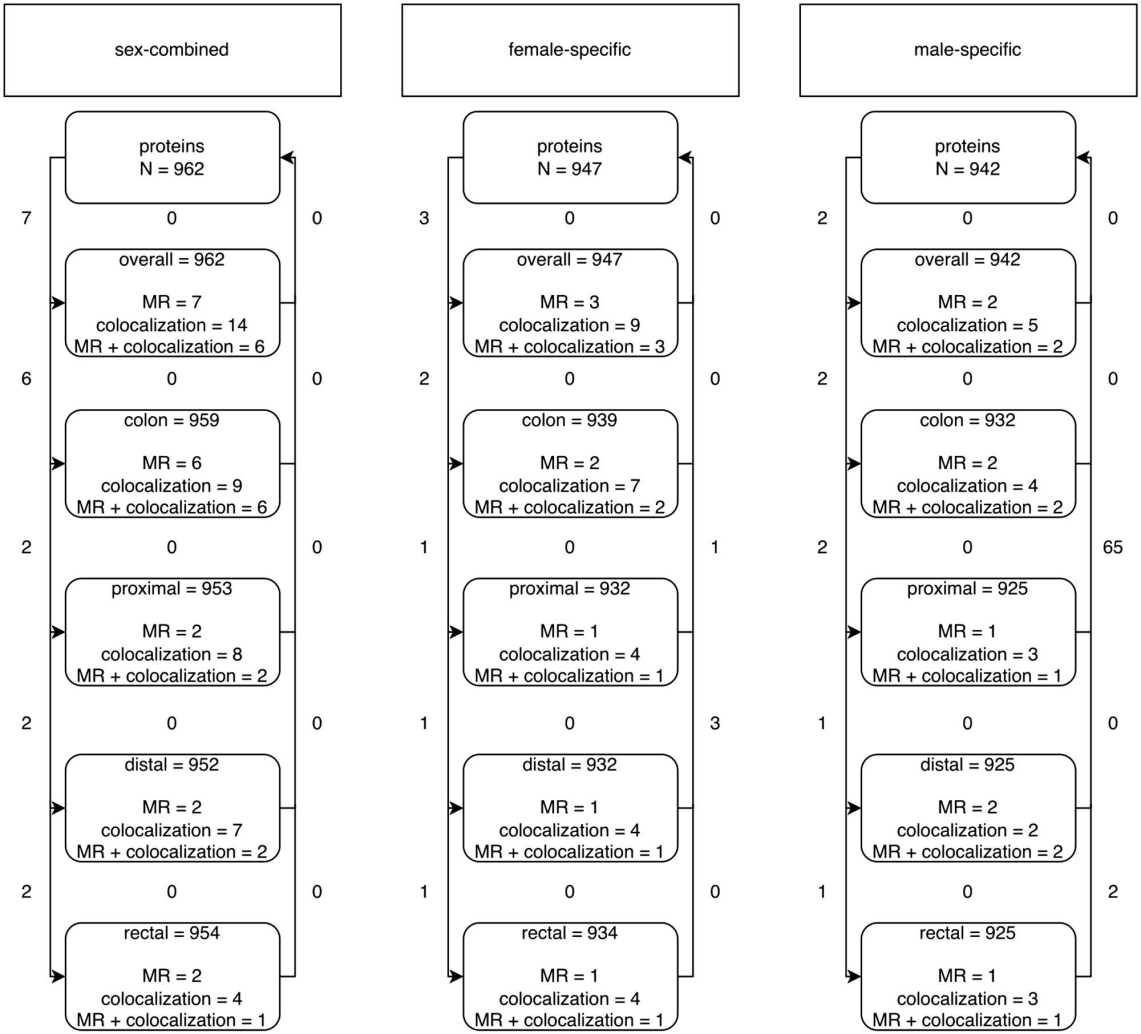
Overview of associations between circulating proteins and colorectal cancer outcomes (Step iii in the main analysis plan). Arrows show the direction of the univariable (UV) Mendelian randomization (MR) (UVMR) analysis (protein–CRC to the left; CRC–protein to the right). Values on the outside of the lines indicate the number of associations identified in that direction; values between the lines indicate the number of associations identified in both directions and for which there is, therefore, conflicting evidence of association. Associations were identified if the PhenoSpD (pheno spectral decomposition) *P*-value threshold (3.97 × 10^−5^) was reached for the forward and reverse MR and if all MR models had consistent directions of effect in the reverse MR. *N* gives the total number of circulating proteins available for analysis. MR gives the number of *cis*-SNP UVMR analyses that reached the PhenoSpD *P*-value threshold for that analysis. Colocalization gives the number of circulating proteins that colocalized with that CRC outcome. MR + colocalization gives the overlap between the *cis*-SNP UVMR and colocalization analyses, and indicates the circulating protein–CRC associations

**Figure 6.**
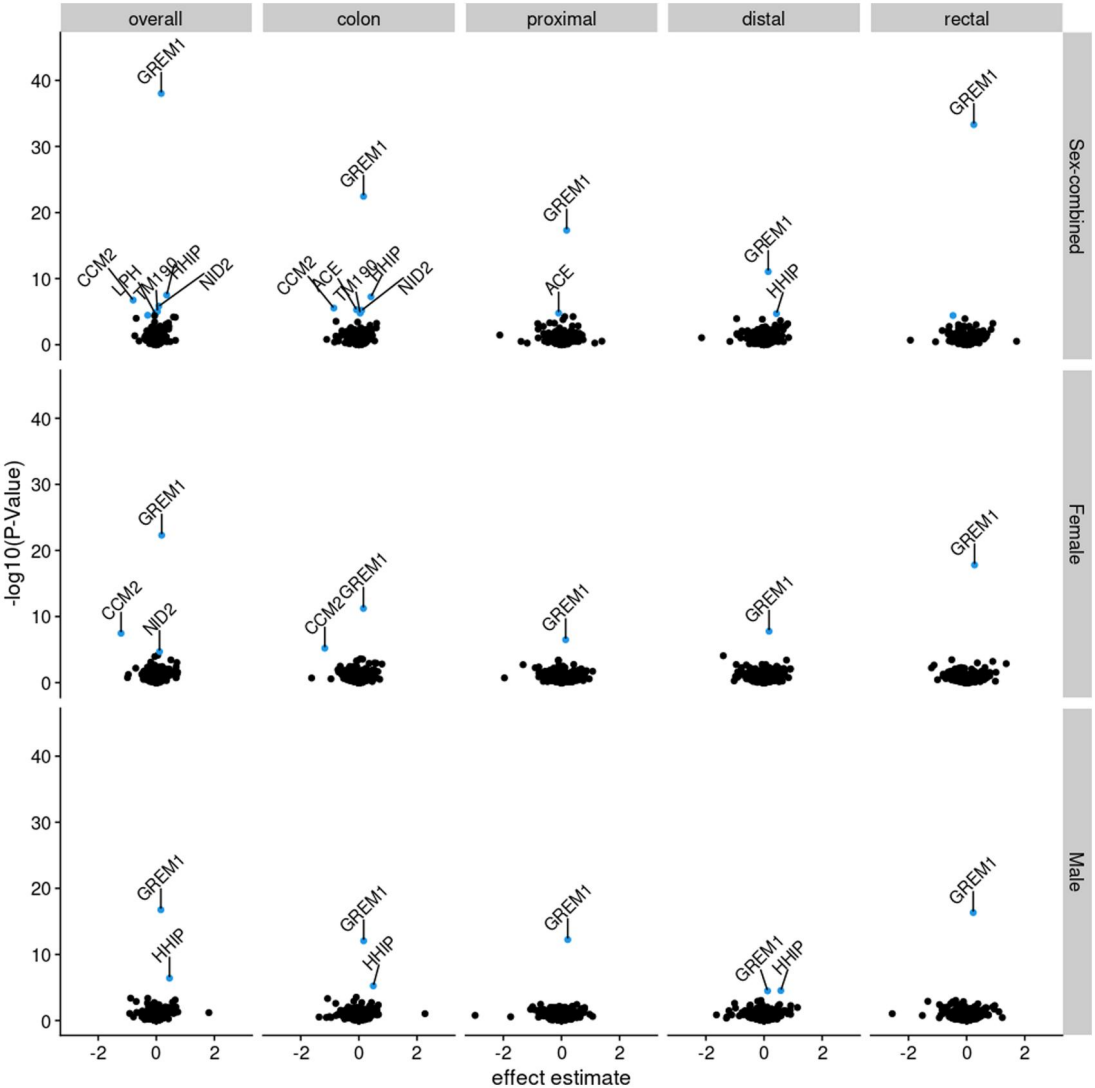
Association between circulating proteins and colorectal cancer outcomes in Step iii of the main analysis plan. The volcano plot shows effect estimates and –log10(pval) with analyses reaching the PhenoSpD corrected *P*-value (0.05/1293) highlighted and analyses reaching the PhenoSpD corrected *P*-value and with evidence of colocalization labeled with the circulating protein name. The *x*-axis has been constrained to –3 to 3, excluding three analyses that did not meet any association thresholds: PTP4A2 and proximal colon cancer in males (effect estimate = 102) and NANS and distal colon cancer in males (effect estimate = 19) and females (effect estimate = 19)

In Step iii, we investigated the association between circulating proteins and CRC outcomes, including analyses by sex and tumor site. Of the 4907 circulating proteins, *cis*-SNPs were available for 1490. Of these, CRC-outcome data were available for ≤962. Overall, 35 protein–CRC associations were identified in the UVMR analyses, involving 8 unique circulating proteins (Figs 5 and 6, and Supplementary Table S6). (See online supplementary material for a color version of this table.) There was evidence (h4 ≥ 0.8) of colocalization for 80 protein–CRC pairs (27 unique circulating proteins; Supplementary Table S7) in our main colocalization analysis (1-mb window; p^1^=10^−6^, p^2^=10^−6^, and p^12^=10^−7^). (See online supplementary material for a color version of this table.) Of these, 66 protein–CRC pairs (22 unique proteins) had evidence across multiple windows and multiple sets of priors. Of the 35 protein–CRC UVMR associations and 66 protein–CRC pairs with evidence across multiple windows and sets of priors, 2 protein–CRC pairs were not corroborated by colocalization (h4 < 0.5): the circulating protein GRFAL in relation to overall CRC and rectal cancer risk in sex-combined analysis. As such, a total of 33 protein–CRC associations (7 unique circulating proteins) were identified by UVMR and corroborated by colocalization analyses (Fig. 5).

### Multivariable Mendelian randomization

**Figure 7.**
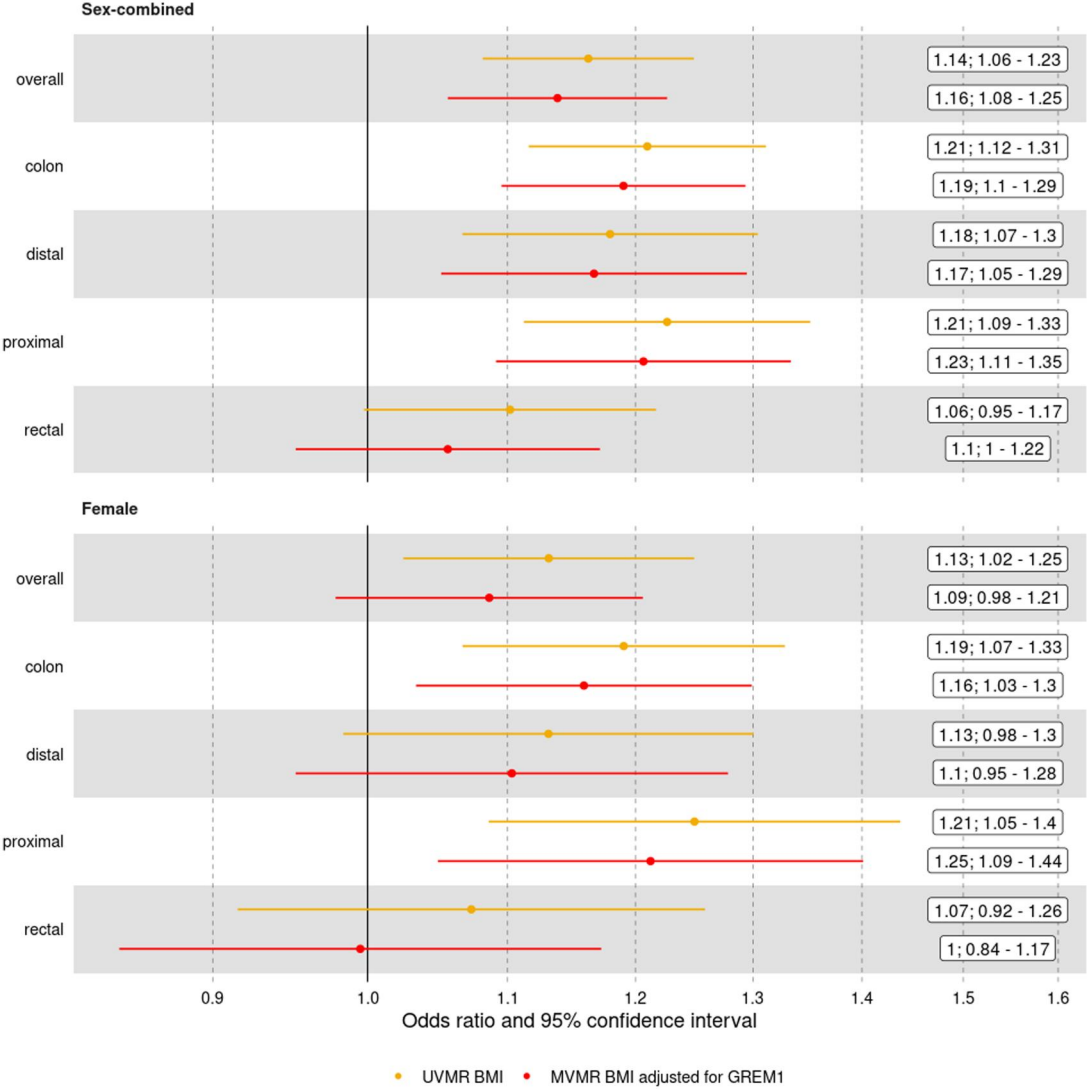
Association between body mass index and colorectal cancer outcomes using univariable (the top estimates) and multivariable (the bottom estimates) Mendelian randomization. In these MVMR analyses (Step iv), the effect of BMI on colorectal cancer outcomes is estimated after adjusting for the effect of GREM1. Odds ratios for the inverse-variance weighted multiplicative random-effects model shown alongside 95% confidence intervals. No adiposity–protein–CRC associations were identified in the male UVMR analyses and, as such, MVMR was not performed

A total of seven circulating proteins were associated with both adiposity (in Step ii of the main analysis plan) and a CRC outcome in UVMR and colocalization analyses (in Step iii), labeled by name in Figs 4 and 6: ACE, CCM2, GREM1, HHIP, LPH, NID2, TM190. Of these, only GREM1 was directionally consistent with a possible mediating role of the association between adiposity and CRC risk (i.e. an increase in genetically predicted BMI was associated with an increase in GREM1 and an increase in genetically predicted GREM1 was associated with an increase in CRC risk). In MVMR (Step iv), we considered a protein as having a potential mediating role if adjusting for the protein attenuated the effect estimate of the adiposity–CRC association toward the null and expanded the 95% confidence interval (CI) to overlap the null, both in comparison with the corresponding UVMR result. For GREM1, effect estimates and CIs for all MVMR analyses tended towards the null compared with UVMR (Fig. 7). However, the CI for the adjusted effect changed from non-overlapping to overlapping the null only in the female-specific analysis of overall CRC risk [OR for BMI in UVMR 1.13 (95% CI = 1.02–1.25); OR after adjustment for GREM1 in MVMR 1.09 (95% CI = 0.98–1.21)]. Conditional F-statistics were >10 (Supplementary Table S8). (See online supplementary material for a color version of this table.) There was evidence that all but the female-specific analysis of distal colon cancer used invalid instruments (Q statistic *P*-value < 0.05; Supplementary Table S8). (See online supplementary material for a color version of this table.)

### Protein expression analyses

**Figure 8.**
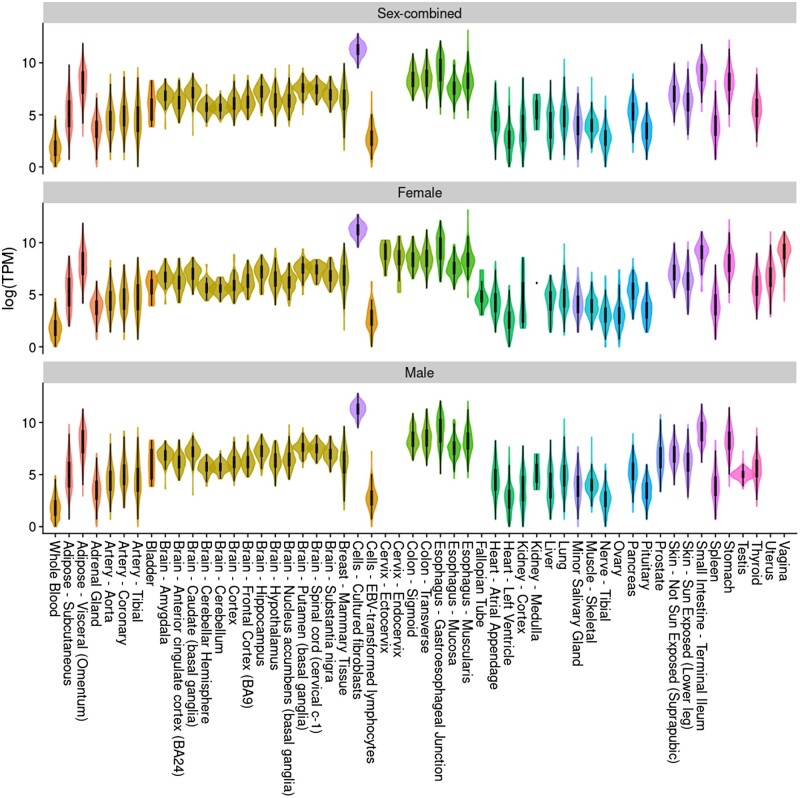
Tissue gene expression profile of GREM1. The violin plot presents expression levels as log transcripts per million (TPM). Data are from GTEx version 8.^32^ Box plots are shown with the interquartile range (25th and 75th percentiles). Different tissue types (e.g. adipose and brain) are highlighted

Using GTEx data, GREM1 was found to be differentially expressed (Bonferroni corrected *P*-value = 0.05/53) in most tissues compared with whole blood, with some of the highest levels noted in the gastrointestinal tract and visceral adipose tissue (Fig. 8).

## Discussion

Using complementary MR and colocalization analyses, we examined the role of the circulating proteome (measured using SomaLogic) as an intermediate in the relationship between measures of overall (BMI) and abdominal/subcutaneous (WHR) adiposity and CRC. We found evidence of a potential mediating role of GREM1 in the association between BMI and overall CRC risk in women. The mediating potential of GREM1 was small and requires further investigation to understand to what extent this is a biologically and/or clinically meaningful reduction.

GREM1, a bone morphogenic protein (BMP) antagonist,^35^ is associated with proliferation, angiogenesis, and epithelial-to-mesenchymal transition of cancer cells.^36^ Studies have linked GREM1 with CRC,^16^^,^^18^^,^^37^^,^^38^ including CRC development^37–39^ and colon cancer tumor progression.^40^ Evidence also links a locus in the *FMN1*/*GREM1* gene region with BMI-related CRC risk.^41^ The mechanisms underlying the GREM1–CRC relationship are unclear but may be related to expression in the tumor microenvironment given GREM1 expression is lower in CRC tissue than in adjacent non-cancerous and normal tissue.^37^ Increased GREM1 expression in CRC tissue has also been associated with low tumor stage and a more favorable prognosis,^42^ and increased GREM1 expression is found in the tumor microenvironment, such as in visceral adipose tissue^43^ and colonic crypt bases via cancer-associated fibroblasts.^44^ Many other circulating proteins showed evidence from MR and/or colocalization analyses and, though they may not be mediators of the adiposity–CRC relationship, they may be markers of this relationship or of CRC risk more broadly.

Adiposity–protein–CRC triples included in the MVMR analyses were supported by consistent directions of effect across the main IVW-MRE model and sensitivity models. We also conducted reverse UVMR analyses for all adiposity–CRC, adiposity–protein, and protein–CRC analyses to identify pairs for which evidence was conflicting. There was some conflicting evidence for the direction of effect in the adiposity–cancer UVMR analyses, although Steiger directionality tests did not indicate that this was likely a result of reverse causation. These conflicts were likely a result of pleiotropy, as indicated by the MR–Egger results, and were therefore excluded from subsequent analyses. Recent work using the same proteomic data set and a different CRC data set highlighted many of the same protein–CRC associations that we observed, including for GREM1.^45^ Our exploratory investigation utilized a single data source for each of our adiposity, protein, and CRC data; triangulating evidence across independent sources of data and study designs is required to strengthen evidence,^46^ and this includes the use of CRC GWASs from prospective studies that would be more robust to issues of reverse causation compared with case–control studies that were predominantly used in the CRC GWAS used here. Only sex-combined data for circulating proteins were available, which may have biased estimates, especially for MVMR analyses in which the exposure and outcome were sex-specific. Larger GWASs of sex- and site-specific CRC, in diverse populations, will be beneficial in further investigating identified effects. Furthermore, we used BMI and WHR as indirect measurements of overall and abdominal/subcutaneous adiposity given the availability of well-powered sex-combined and sex-specific GWASs. Instrument strength, measured via F-statistics, was appropriate for most analyses; however, there was evidence of weak instrument bias across most MVMR analyses, which can lead to estimates for the exposure and intermediate moving towards and away from the null.^30^ The SomaLogic platform measures a defined set of proteins and SNPs were available for fewer than half of those measured.^18^ Evidence from GTEx showed that GREM1 gene expression is upregulated in gastrointestinal and adipose tissue compared with whole blood, which may suggest that results using circulating GREM1 reflect exposure levels in relevant tissues.

Our results highlight that many adiposity-associated circulating proteins are also associated with the risk of CRC. We found evidence of one adiposity-related circulating protein, GREM1, as a likely mediator of the adiposity and CRC relationship, particularly for women. Our results suggest that the GREM1 pathway may be a potential mechanism underlying the adiposity–CRC relationship. Future replication and experimental investigation to inform on tissue-specific effects of these proteins using independent data sets and different proteomic platforms is warranted.

## Disclaimer

Where authors are identified as personnel of the International Agency for Research on Cancer/World Health Organization, the authors alone are responsible for the views expressed in this article and they do not necessarily represent the decisions, policy, or views of the International Agency for Research on Cancer/World Health Organization. This article is the result of the scientific work of Dr Murphy while he was affiliated at the International Agency for Research on Cancer.

## Ethics approval

This study used summary-level data; as such, ethical approval was not sought. For adiposity data, UK Biobank has approval from the North West Multi-centre Research Ethics Committee as a Research Tissue Ban; this means that researchers do not require separate ethical clearance and can operate under the Research Tissue Bank approval. For proteomic data, all participants who donated samples gave informed consent, and the National Bioethics Committee of Iceland approved the study, which was conducted in agreement with conditions issued by the Data Protection Authority of Iceland (VSN_14-015). For CRC data, all participants provided written informed consent, and each study was approved by the relevant research ethics committee or institutional review board.

## Supplementary Material

dyae175_Supplementary_Data

## Data Availability

This work is supported by a GitHub repository (https://github.com/mattlee821/adiposity_proteins_colorectal_cancer), which is archived on Zenodo (https://zenodo.org/record/7780822#.ZCQ3U-xBz0o). Here, all publicly available data, code, and results used in this work are available. The full summary statistics for BMI and WHR are publicly available from Zenodo (https://zenodo.org/record/1251813#.Yk7O25PMIUE). The full summary statistics for all proteins are publicly available from DECODE (https://download.decode.is/form/folder/proteomics). The full summary statistics for CRC are not publicly available but can be obtained from GECCO (https://www.fredhutch.org/en/research/divisions/public-health-sciences-division/research/cancer-prevention/genetics-epidemiology-colorectal-cancer-consortium-gecco.html). All results generated from the analyses presented in this work are available as supplementary data.
