# Supplementary material for: A proteogenomic analysis of the adiposity colorectal cancer relationship identifies GREM1 as a probable mediator
Source: Int J Epidemiol. 2025 Jan 22;54(1):dyae175. doi: 10.1093/ije/dyae175 (PMC11754674; doi:10.1093/ije/dyae175)

**Supplementary Figures**

**Figure S1. Association between adiposity measures and colorectal cancer outcomes.**

Effect estimates and 95% confidence intervals shown for the main analysis using the inverse variance weighted multiplicative random effects (IVW-MRE) model and 3 sensitivity models.

BMI = body mass index; WHR = waist-hip ratio.


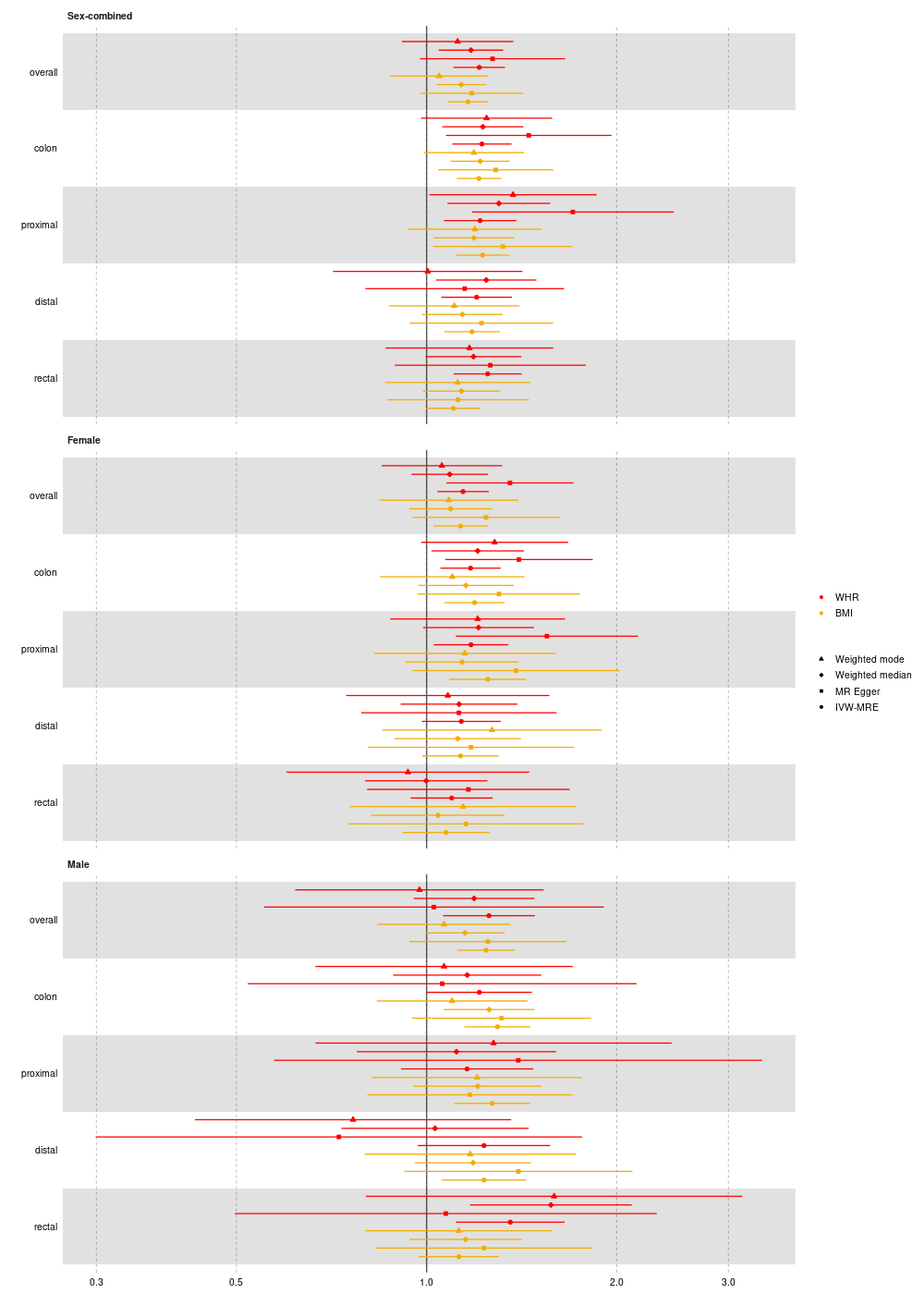


**Figure S2. Association between colorectal cancer measures and adiposity measures.**

Effect estimates and 95% confidence intervals shown for the main analysis using the inverse variance weighted multiplicative random effects (IVW-MRE) model and 3 sensitivity models.

BMI = body mass index; WHR = waist-hip ratio.


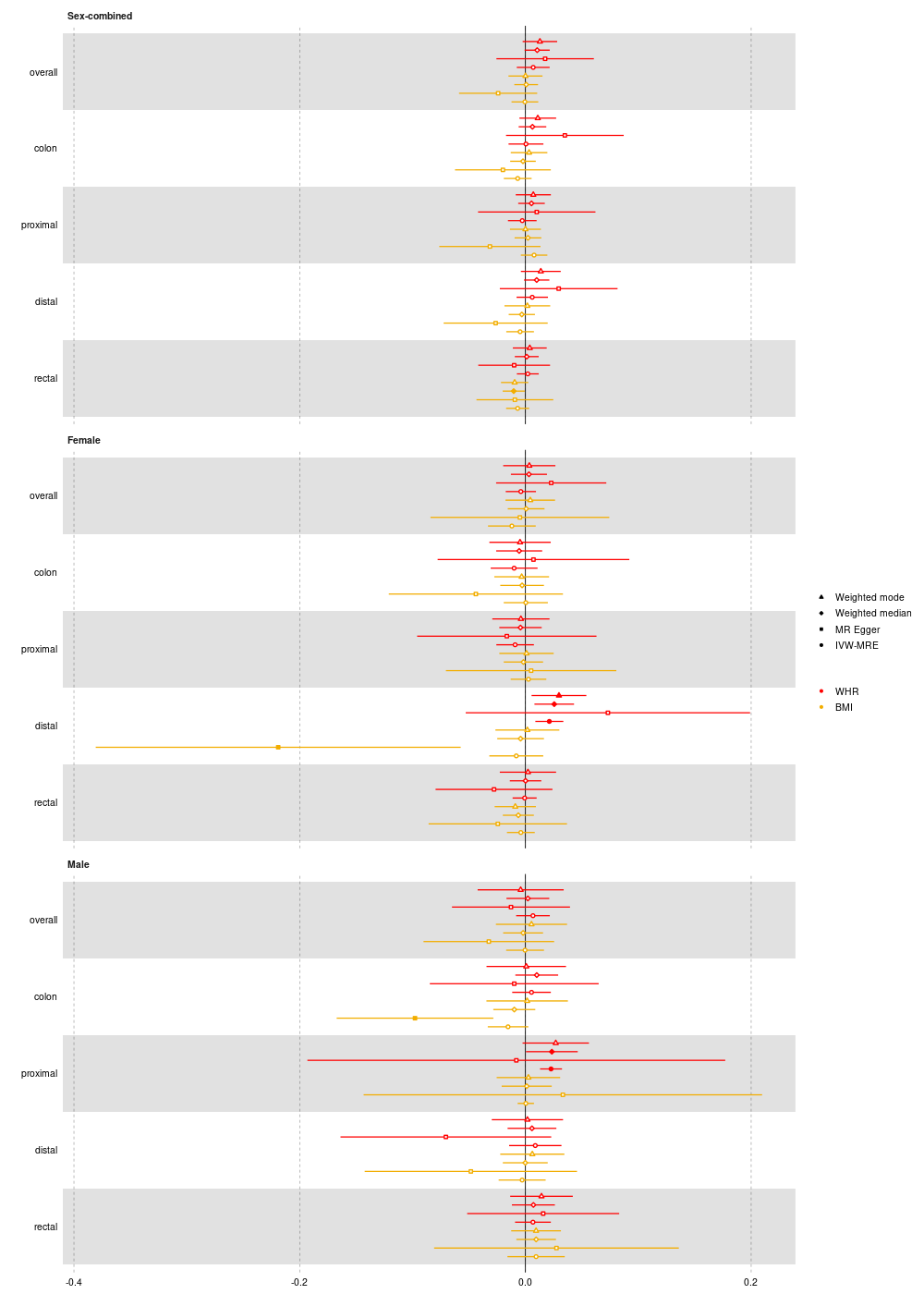

Supplement: dyae175_Supplementary_Data [file dyae175_supplementary_data.zip › ije-2023-08-1026-File016.docx]
